# The cryo‐EM structure of full‐length RAD52 protein contains an undecameric ring

**DOI:** 10.1002/2211-5463.13565

**Published:** 2023-02-09

**Authors:** Chiaki Kinoshita, Yoshimasa Takizawa, Mika Saotome, Shun Ogino, Hitoshi Kurumizaka, Wataru Kagawa

**Affiliations:** ^1^ Department of Chemistry, Graduate School of Science and Engineering Meisei University Tokyo Japan; ^2^ Laboratory of Chromatin Structure and Function, Institute for Quantitative Biosciences The University of Tokyo Japan

**Keywords:** cryo‐electron microscopy, DNA annealing protein, DNA double‐strand break repair, intrinsically disordered region, oligomerisation, ring structure

## Abstract

The human RAD52 protein, which forms an oligomeric ring structure, is involved in DNA double‐strand break repair. The N‐terminal half of RAD52 is primarily responsible for self‐oligomerisation and DNA binding. Crystallographic studies have revealed the detailed structure of the N‐terminal half. However, only low‐resolution structures have been reported for the full‐length protein, and thus the structural role of the C‐terminal half in self‐oligomerisation has remained elusive. In this study, we determined the solution structure of the human RAD52 protein by cryo‐electron microscopy (cryo‐EM), at an average resolution of 3.5 Å. The structure revealed an undecameric ring that is nearly identical to the crystal structures of the N‐terminal half. The cryo‐EM map for the C‐terminal half was poorly defined, indicating that the region is intrinsically disordered. The present cryo‐EM structure provides important insights into the mechanistic roles played by the N‐terminal and C‐terminal halves of RAD52 during DNA double‐strand break repair.

## Graphical abstract

The cryo‐EM structure of the human DNA repair protein RAD52 was determined at near‐atomic resolution, which revealed that the full‐length protein oligomerises into an undecameric ring. The N‐terminal half is responsible for the oligomerisation. By contrast, the C‐terminal half was not visible in the structure, which is consistent with the predicted intrinsic disorder of the region.

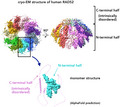

**Abbreviations**
BIR: break‐induced replication
cryo‐EM: cryo‐electron microscopy
CTF: contrast transfer function
DSBR: double‐strand break repair
DSBs: DNA double‐strand breaks
FSC: Fourier Shell Correlation
HDR: homology‐directed repair
RPA: replication protein A
SDSA: synthesis‐dependent strand annealing
SSA: single‐strand annealing
SSAP: single‐strand annealing protein
ssDNA: single‐stranded DNA

Genomic DNA is constantly damaged by endogenous factors, such as replication errors and reactive oxygen species generated as by‐products of normal oxygen metabolism [1, 2]. These DNA damaging factors can cause DNA double‐strand breaks (DSBs), which are the most lethal type of DNA damage. Accurate repair of DSBs is important for preserving genome integrity and avoiding cancer. Cells utilise undamaged homologous sequences as templates for accurate repair [3]. There are several DSB repair pathways that rely on the presence of homologous sequences, and they are collectively termed homology‐directed repair (HDR) [4, 5]. Homology‐directed repair includes double‐strand break repair (DSBR), synthesis‐dependent strand annealing (SDSA), break‐induced replication (BIR) and single‐strand annealing (SSA). Although the RAD51 protein plays a central role in many of the HDR pathways, increasing evidence suggests that RAD51‐independent HDR pathways may also have significant roles, in events such as alternative telomere maintenance and mitotic DNA synthesis. The molecular mechanisms of these repair pathways, however, are still largely unknown.

RAD52 is considered to function in multiple HDR pathways. In yeast, Rad52 functions as a recombination mediator in DSBR, by facilitating the assembly of the Rad51 recombinase on replication protein A (RPA)‐coated ssDNA [6, 7]. Rad52 is also a key player in SSA and promotes the annealing of complementary DNA strands [8, 9]. In humans, it is not clear whether RAD52 functions as a mediator of RAD51 [10]. However, like its yeast counterpart, RAD52 stimulates DNA annealing [11]. Human RAD52 is also capable of catalysing the formation of D‐loops *in vitro* [12], which may be a relevant activity in certain RAD51‐independent HDR pathways. More recently, RAD52 was suggested to function in mitotic DNA synthesis and alternative lengthening of telomeres, via RAD51‐independent mechanisms [13, 14, 15, 16]. Given the multitude of roles played by RAD52, uncovering its precise functions in these repair pathways could lead to a more comprehensive understanding of the mechanisms of HDR.

From an evolutionary perspective, RAD52 belongs to the single‐strand annealing protein (SSAP) superfamily, whose members share a common function of promoting the annealing of complementary DNA strands during DNA repair. Several members of this superfamily, including Erf, RecT, Sak, ICP8, DdrB and Redβ, reportedly oligomerise into rings and filaments [17, 18, 19, 20, 21, 22]. Mechanistic models have been proposed in which the quaternary structures of these proteins dynamically change during the annealing of complementary DNA strands [23]. Yeast and human RAD52 also form oligomeric rings [24, 25]. Although the amino acid sequence similarity is low between RAD52 and the other SSAPs, it is plausible that the quaternary structure of RAD52 may also play an important role during DSB repair.

Crystallographic studies of the N‐terminal fragment of RAD52 have provided the structural framework for understanding its detailed functions. The N‐terminal half of RAD52 is highly conserved among RAD52 homologs and is primarily responsible for self‐association, DNA binding and DNA annealing [26, 27, 28]. Fragments of the N‐terminal half of RAD52 oligomerise into a ring structure containing 11 protomers. Structure‐based mutagenesis studies have successfully determined the amino acid residues that are necessary for the DNA binding and annealing activities of the full‐length protein [26, 28, 29, 30]. While the crystal structures of the N‐terminal fragment have been useful for identifying the amino acid residues important for the function of the full‐length protein, it is unknown whether the tertiary and quaternary structures formed by the N‐terminal fragment are preserved in the full‐length protein.

The full‐length RAD52 protein has been visualised in several previous studies by negative staining electron microscopy [25, 31]. However, the resolution in these studies was not sufficient to allow detailed comparisons with the crystal structures, and to provide clues as to which amino acid residues play key roles in the function of RAD52. To gain further insights into the structure of the full‐length RAD52 protein, we determined its solution structure by cryo‐electron microscopy (cryo‐EM) at near‐atomic resolution. The structure revealed an undecameric assembly that is nearly identical to that observed in the crystal structures of the N‐terminal fragment.

## Materials and methods

### Expression and purification of RAD52 for cryo‐EM studies

The expression and purification procedures for the human RAD52 protein (UniProt‐ID P43351) were adopted from those described in previous biochemical studies [12, 28], with some modifications. Briefly, RAD52 was overexpressed in the *Escherichia coli* strain JM109(DE3) as an N‐terminally hexahistidine‐tagged protein. To enhance the expression of RAD52, tRNAs that recognize rare arginine codons, CGG, AGA and AGG, were co‐expressed using the pArg3Arg4 vector [12]. Freshly grown colonies were directly transferred to LB medium (800 mL) containing 100 μg·mL^−1^ ampicillin and 34 μg·mL^−1^ chloramphenicol, and cultured at 30 °C. RAD52 expression was induced with isopropyl 1‐thio‐β‐d‐galactopyranoside (0.5 mm final concentration), when the optical density (A_600_) of the medium reached 0.6. The culture was continued for 16 h. Cells were collected by centrifugation, resuspended in buffer A (50 mm Tris–HCl, pH 7.8, 0.3 m KCl, 5 mm 2‐mercaptoethanol, and 10% glycerol) containing 10 mm imidazole, and lysed by sonication. The resulting cell lysate was centrifuged to remove insoluble material.

The soluble fraction was mixed with Ni‐NTA agarose beads (2 mL) that were prewashed with buffer A containing 10 mm imidazole, and gently rotated for 15 min at 4 °C. The mixture was then poured into an Econo‐column. The packed Ni‐NTA column was washed with 60 mL of buffer A containing 50 mm imidazole. RAD52 was eluted with a 60‐mL linear gradient of 50–400 mm imidazole in buffer A. Peak fractions were pooled, and 3 units of thrombin protease per mg of protein were added to cleave the hexahistidine tag. The pooled fractions were immediately dialysed against buffer B (20 mm HEPES‐KOH, pH 7.5, 0.5 mm EDTA, 2 mm 2‐mercaptoethanol, and 5% glycerol) containing 0.2 m KCl. After confirming the cleavage of the hexahistidine‐tag by SDS/PAGE, the dialysed sample was loaded onto a 2.1 mL SP Sepharose column that was pre‐equilibrated with buffer B containing 0.2 m KCl. The column was washed with 60 mL of buffer B containing 0.2 m KCl, and RAD52 was eluted with a 60‐mL linear gradient of 0.2–0.8 m KCl in buffer B. Peak fractions were pooled, and concentrated to approximately 5 mg·mL^−1^ with a Vivaspin Turbo 15 centrifugal filter (100 K MWCO). The concentrated fraction was loaded onto an ENrich SEC 650 column (Bio‐Rad, Hercules, CA, USA) that was pre‐equilibrated with buffer C (20 mm HEPES‐KOH, pH 7.5, 0.1 mm EDTA, 2 mm 2‐mercaptoethanol, and 0.4 m KCl). Peak fractions were separately stored on ice, and used for cryo‐EM specimen preparation within 24 h. The concentration of RAD52 was determined from the absorbance at 280 nm, using an extinction coefficient of 39 880 m ^−1^ cm^−1^ calculated with the Protparam tool on the ExPASy website (https://web.expasy.org/protparam/).

### Cryo‐EM specimen preparation for RAD52

A 2.5 μL portion of the purified RAD52 sample (~ 0.4 mg·mL^−1^) was applied onto a freshly glow‐discharged holey carbon grid (Quantifoil R1.2/1.3, Cu, 200 mesh). Using a Vitrobot Mark IV (Thermo Fisher Scientific, Waltham, MA, USA), the grid was blotted for 6 s at 4 °C in 100% humidity, and then plunge‐frozen in liquid ethane.

### Cryo‐EM data collection

The full‐length RAD52 protein was imaged on a Krios G4 microscope (Thermo Fisher Scientific), operated at 300 kV and equipped with an energy‐filtered K3 direct electron detector with a slit width of 20 eV, operating in the counting mode at a calibrated pixel size of 1.06 Å. Movies of the full‐length RAD52 were recorded at a frame rate of 150 ms for 4.5 s, for a total accumulated dose of 57.6 electrons per Å^2^. A nominal defocus range of 1–2.5 μm was employed, and the movies were automatically acquired using the epu software (Thermo Fisher Scientific).

### Image processing and 3D reconstruction of RAD52

All frames of the 5044 movies were aligned using motioncor2 with dose weighting [32], and the contrast transfer function (CTF) was estimated using ctffind4 [33]. relion3.1 was used for the following image processing of full‐length RAD52 [34]. A total of 2 860 807 particles were picked from 3789 micrographs by Template‐based auto‐picking, using the 2D class averages of auto‐picked particles based on a Laplacian‐of‐Gaussian filter as templates, followed by two rounds of 2D classification to remove junk particles, resulting in the selection of 1 550 950 particles. The crystal structure of RAD52 ^1–212^ (PDB ID: 1KN0) was low‐pass filtered to 60 Å and used as the initial model for the 3D classification. The selected particles were subjected to a few rounds of 3D classifications. Subsequently, the best class from the 3D classification, containing 39 100 particles, was subjected to 3D refinement with C11 symmetry, Bayesian polishing and CTF refinement. The final postprocessing yielded a cryo‐EM map with a global resolution of 3.48 Å, which was estimated from the gold standard Fourier Shell Correlation (FSC = 0.143) criteria [35]. The local resolution of the full length RAD52 was estimated by relion‐3.1. The cryo‐EM map of the full‐length RAD52 was normalised with mapman [36].

### Model building and refinement

Model building was performed with coot [37], using the crystal structure of the N‐terminal half as the initial model (PDB ID: 1KN0) [26]. The model was refined by multiple rounds of real‐space refinement in phenix [38, 39]. Structural validation of the resulting model was performed with molprobity [40]. The statistics associated with the 3D reconstruction and model refinement are shown in Table 1. Molecular graphics generation and solvent‐accessible surface area calculations were performed with ucsf chimerax [41].

**Table 1:** Data collection and refinement statistics.

Data acquisition	
Microscope	Krios G4
Voltage (kV)	300
Magnification	81 000
Detector	K3
Pixel size (Å)	1.06
Electron exposure (e^−^/Å^2^)	57.6
Defocus range (μm)	−1 to −2.5 μm
Image processing	
EMDB accession ID	EMD‐34430
Initial number of particles	2 860 807
Final number of particles	39 100
Symmetry	C11
Map resolution (Å)	3.48
Map resolution range (Å)	3.46 to 6.07 Å
Refinement	
Initial model (PDB ID)	1KN0
Model resolution (Å)	3.5
FSC_model vs. map_ = 0.5 (Å)	3.5
Model‐map correlation coefficient	
Mask	0.79
Box	0.71
Peaks	0.69
Volume	0.77
Model	
PDB accession ID	8H1P
Number of nonhydrogen atoms	15 950
Number of protein residues	2 024
*B* factor (Å^2^)	69.07
Root‐mean‐square deviation	
Bond length (Å)	0.003
Bond angle (°)	0.559
Validation	
molprobity score	1.52
molprobity clash score	10.09
Rotamer outliers (%)	0
C_β_ outliers (%)	0
Ramachandran plot (%)	
Favored	99.45
Allowed	0.55
Outliers	0

## Results

### Cryo‐EM structure of RAD52

**Fig. 1:**
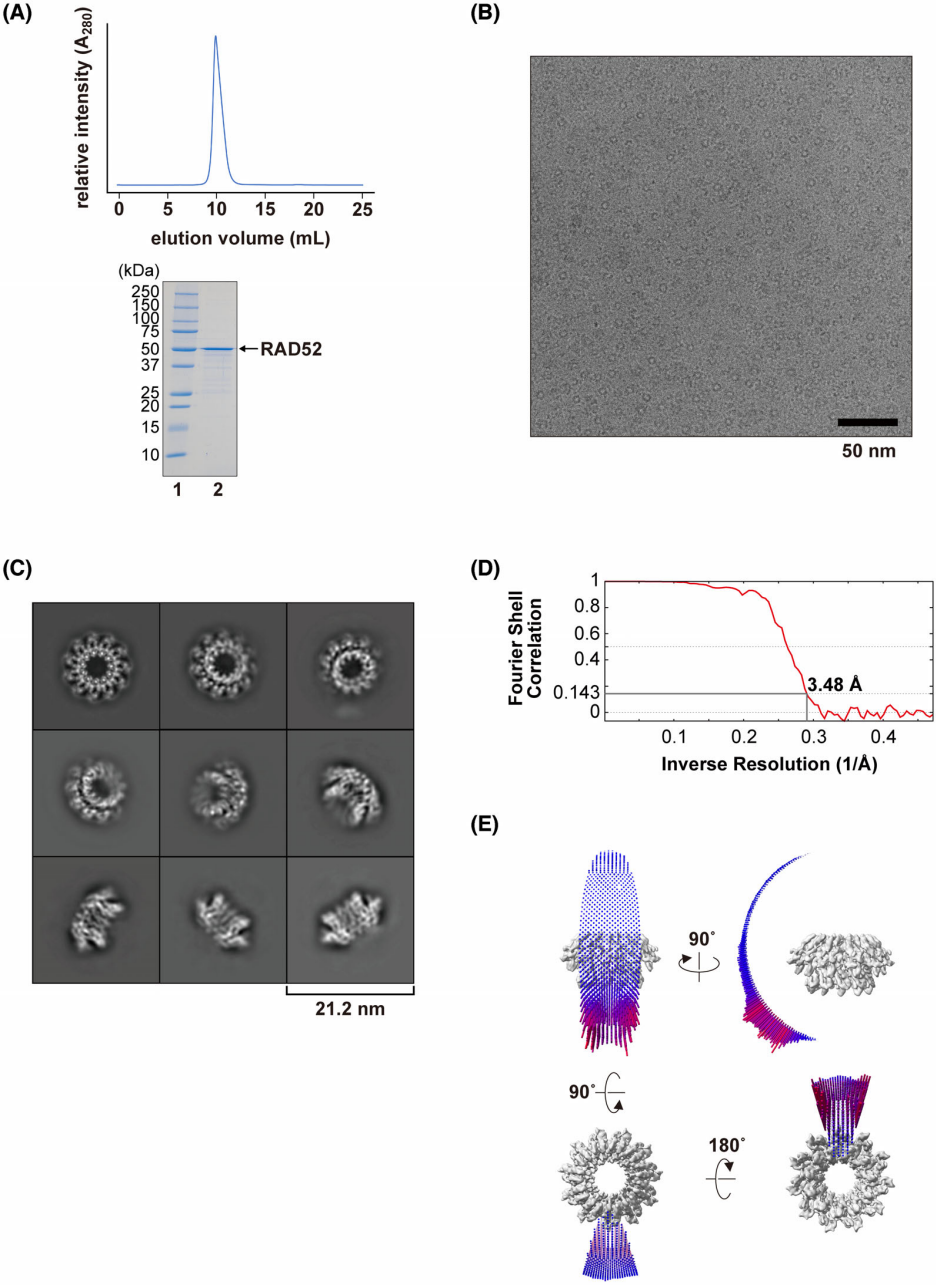
Cryo‐EM analysis of the full‐length RAD52 protein. (A) Purification of RAD52. Size exclusion chromatography of RAD52 (top). RAD52 eluted from the gel filtration column as a single peak, with an elution volume of approximately 10 mL. The peak fraction (~ 0.4 mg·mL^−1^) used for cryo‐EM analysis, was fractionated by a 10–20% gradient polyacrylamide gel electrophoresis (bottom). A 1 μg portion of the purified RAD52 was visualized by Coomassie Brilliant Blue staining (lane 2). Molecular weight markers indicate 250, 150, 100, 75, 50, 37, 25, 20, 15, and 10 kDa from top to bottom (lane 1). (B) A representative cryo‐EM micrograph showing RAD52 particles. The scale bar represents 50 nm. (C) Representative 2D class averages of RAD52 were calculated from the cryo‐EM dataset. The box width corresponds to 21.2 nm. (D) FSC curve for the 3D reconstruction of RAD52. The FSC of 0.143 indicates a resolution of 3.48 Å. (E) Euler angle distribution of all particles used in the final map reconstruction. Each particle orientation is represented by a cylinder. The height and color (blue to red) of the cylinder are proportional to the number of particles.

**Fig. 2:**
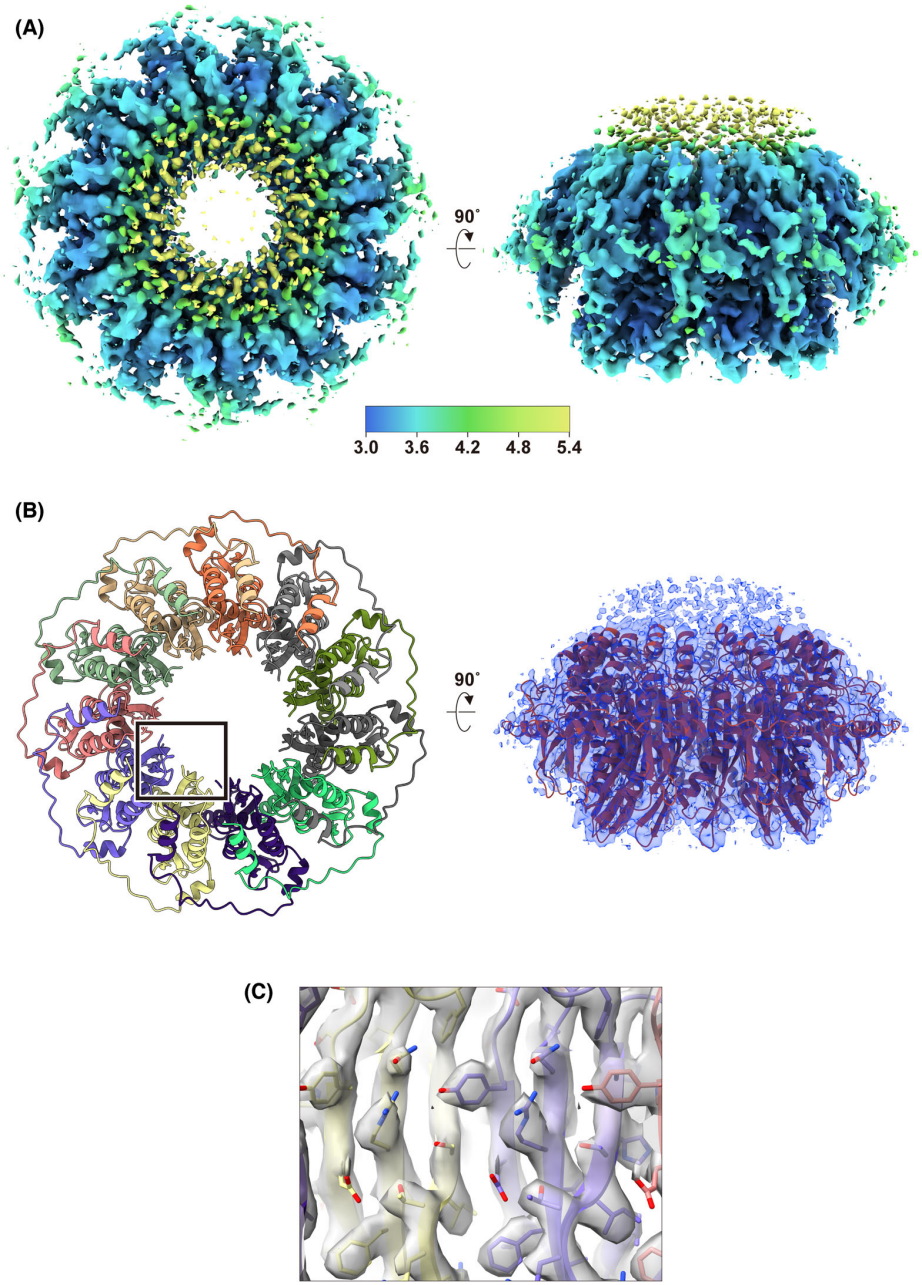
Refined cryo‐EM map and atomic model of RAD52. (A) Top (left) and side (right) views of the reconstructed 3D map of RAD52, colored by local resolution (ranges from 3.0 to 5.4 Å). (B) (Left) Ribbon representation of the refined model, viewed down the central channel. Each protomer is colored differently. (Right) The refined model (light red) superimposed with the cryo‐EM map (light blue). (C) A close‐up view of the inner wall of the central channel (boxed region in B).

To determine the full‐length structure of the human RAD52 protein, the freshly prepared protein was fractionated by size‐exclusion chromatography (Fig. 1A), and peak fractions were directly imaged by cryo‐EM. RAD52 has an intrinsic tendency to self‐aggregate at increased concentrations, and thus protein concentration prior to imaging was avoided. We also note that KCl concentrations higher than 0.2 m were effective in preventing RAD52 aggregation, and thus the final buffer included 0.4 m KCl. For the grid preparation, we tested several RAD52 concentrations, and found that approximately 0.4 mg·mL^−1^ of RAD52 resulted in well‐dispersed images of ring structures (Fig. 1B). A total of 2 860 807 particles were extracted from 3789 micrographs, from which 2D class averages were generated. In many of the 2D class averages, 11 protomers were clearly distinguishable in the apparent oligomeric ring structure (Fig. 1C). The final 3D cryo‐EM map of RAD52 was refined to a resolution of 3.48 Å (Fig. 1D), and no strongly preferred orientation was observed (Fig. 1E). The map revealed a mushroom‐like structure (Fig. 2A), much like the crystal structure of the N‐terminal half determined previously [26, 27].

**Fig. 3:**
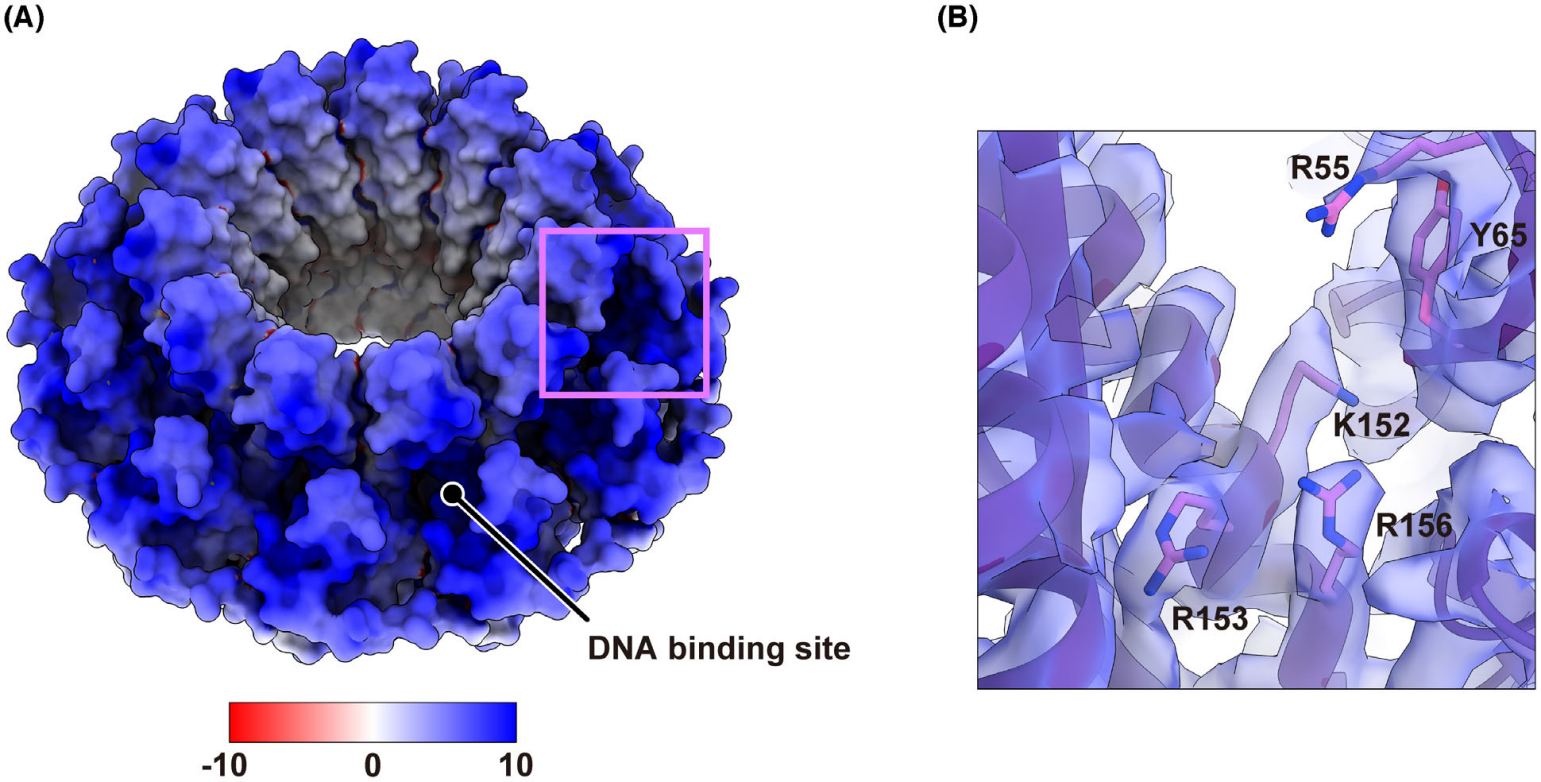
DNA binding site of RAD52. (A) Solvent‐accessible surface of RAD52, colored according to the electrostatic potential from −10 *k*_*B*_ *Te*_*c*_^−1^ (red) to 10 *k*_*B*_ *Te*_*c*_^−1^ (blue). The electrostatic potential of the atomic model was calculated using the Adaptive Poisson‐Boltzmann Solver (APBS) web server (https://server.poissonboltzmann.org) [44]. (B) Close‐up view of the DNA binding site (magenta box shown in (A)) composed of Arg55, Tyr65, Lys152, Arg153, and Arg156. The cryo‐EM map (light blue) is superimposed with the atomic model.

An atomic model of RAD52 was constructed by fitting the crystal structure of the N‐terminal half (PDB ID: 1KN0) into the cryo‐EM map, followed by iterative rounds of model adjustment and refinement (Fig. 2B, left). The final model fit well into the cryo‐EM map (chimera correlation coefficient of 0.69; Fig. 2B, right) with acceptable geometry (molprobity score 1.52, Ramachandran favored 99.45% and no outliers; Table 1). Many of the bulky side chains located at or near the central channel of the ring structure were clearly visible in the cryo‐EM map (Fig. 2C). The successful fitting of the crystal structure of the N‐terminal half into the cryo‐EM map indicates that the undecameric ring structure observed for the N‐terminal RAD52 fragment is conserved in the full‐length protein. The narrow DNA binding groove detected in previous crystallographic studies is also conserved in the present cryo‐EM structure, along with the electron potential maps of key DNA binding residues (Fig. 3) [26, 28, 30]. These observations suggest that the mechanisms for DNA binding and DNA annealing proposed from the studies of the N‐terminal RAD52 fragment are applicable for the full‐length protein.

### Location of the C‐terminal half

**Fig. 4:**
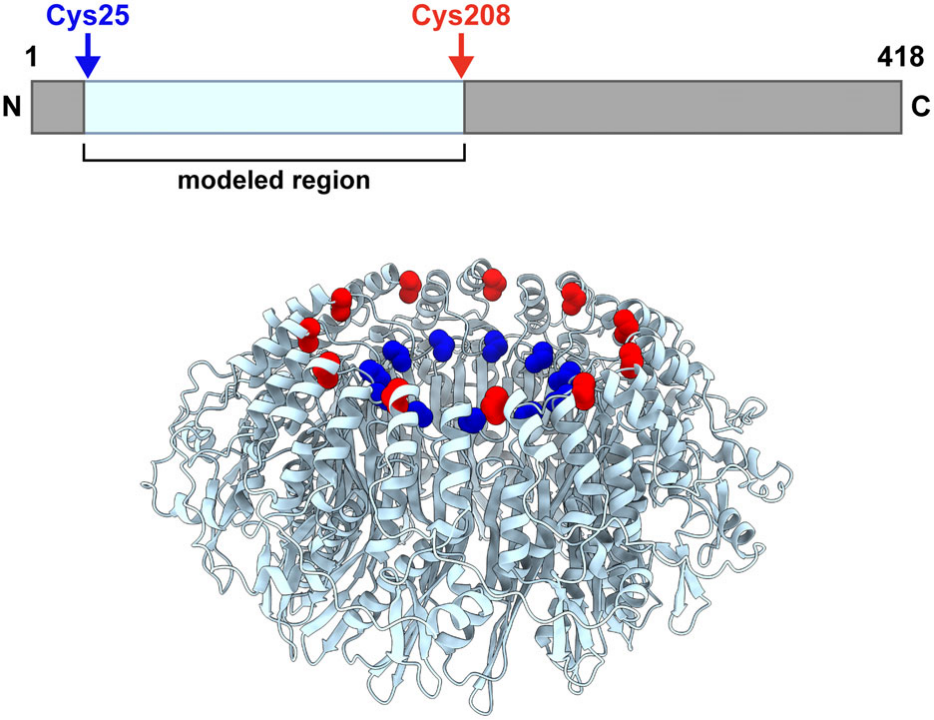
Locations of the N‐ and C‐terminus of the refined RAD52 structure with respect to the cryo‐EM map. (Top) The modeled region (RAD52^25–208^, pale cyan) is from the N‐terminal half of RAD52. (Bottom) The N‐terminus (Cys25, blue) and the C‐terminus (Cys208, red) are clustered near the top of the ‘domed cap’ region of the oligomeric ring structure.

A fragmented map was observed at the top of the mushroom‐like structure (Fig. 2A,B). The fitted model did not occupy those regions, suggesting that they are attributable to the N‐terminal 24 amino acid residues and the C‐terminal half of RAD52 (RAD52^209–418^), which were both absent in the initial model (Fig. 4). The N‐terminus (Cys25) and the C‐terminus (Cys208) of the fitted model are located nearby (Fig. 4). We attempted to build a model for these regions, based on the cryo‐EM map. However, the local resolution of the unoccupied map was relatively low (~ 5 Å; Fig. 2A), and model building was unsuccessful due to the lack of detailed structural information. Thus, the N‐terminal 24‐amino acid region and the C‐terminal half of RAD52 likely have flexible structures, and are probably located at the top of the mushroom‐like structure.

### Comparison with the crystal structure of RAD52

**Fig. 5:**
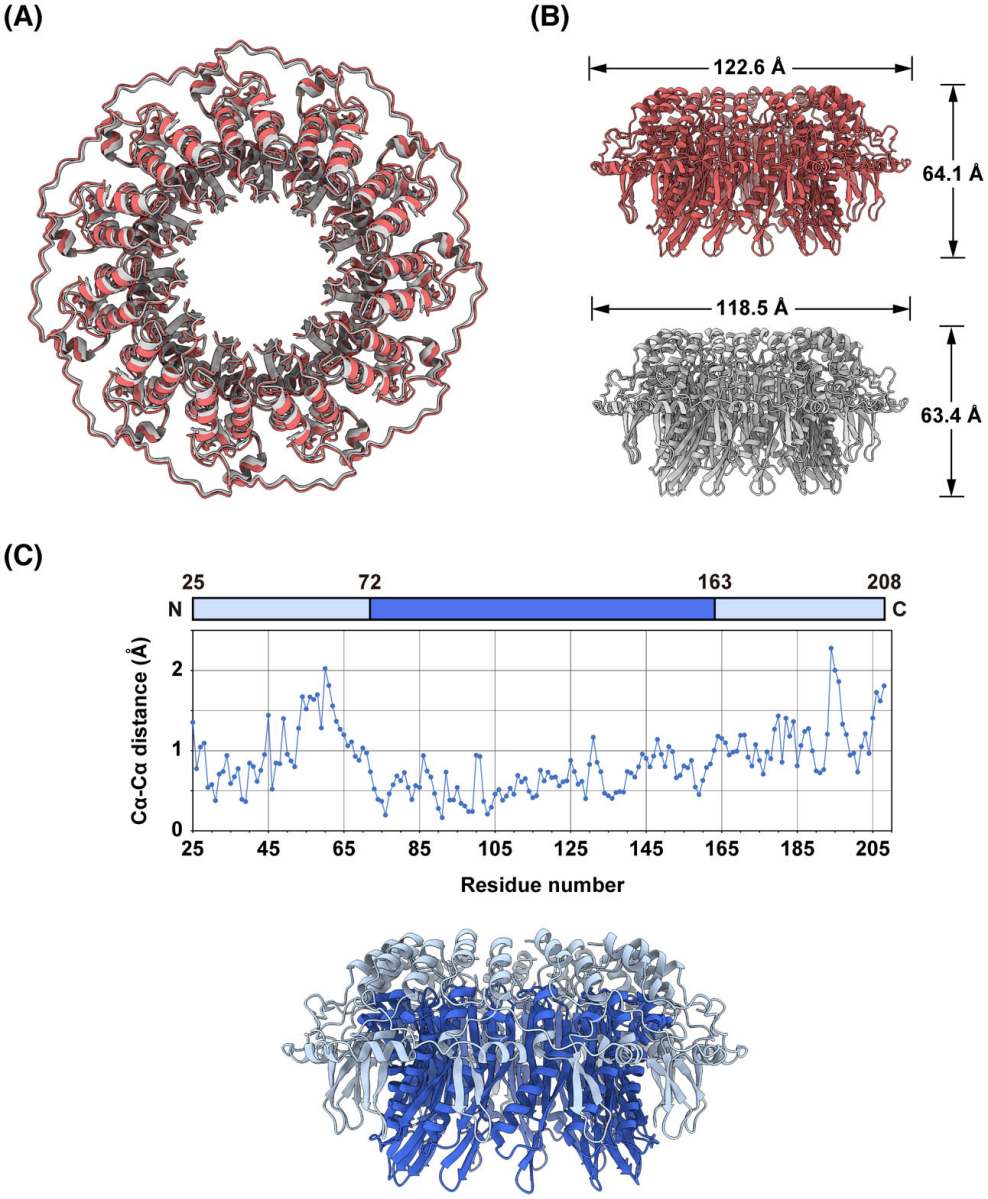
Comparison between the cryo‐EM and crystal structures of RAD52. (A) Superimposition of the cryo‐EM structure (light pink) with the crystal structure of the N‐terminal half (light gray; PDB ID: 1KN0). The align command in pymol (Schrödinger, LLC., New York, NY, USA) was used for the superimposition and the calculation of the overall root mean square deviation. (B) Orthoscopic side views of the cryo‐EM structure (top), and the crystal structure (bottom; PDB ID: 1KN0). The diameters and heights of the ring structures are indicated. The values were calculated with pymol, using the Draw_Protein_Dimensions.py script. (C) Graphical representation of the Cα‐Cα distances between the superimposed structures (top). The N‐terminal (amino acid residues 25–71) and C‐terminal (amino acid residues 164–208) regions with relatively longer Cα–Cα distances correspond to the ‘domed cap’ region (pale cyan; bottom). The central region (amino acid residues 72–163) corresponds to the ‘stem’ region (blue).

The cryo‐EM structure is highly similar to the crystal structure of the N‐terminal half, as shown in the structural alignment (Fig. 5A). The root mean square deviation between the two structures is 0.981 Å. A closer inspection, however, revealed notable differences. The solvent‐accessible surface area of the cryo‐EM structure (87 647 Å^2^) is significantly larger than that of the crystal structure (79 584 Å^2^), indicating that the cryo‐EM structure exhibits a more loosely folded structure. This is apparent when the superimposed structures are viewed down the rotational symmetry axis running through the central channel of the ring (Fig. 5A). The cryo‐EM structure is slightly expanded outward, as compared to the crystal structure. The diameter of the cryo‐EM structure (122.6 Å) is marginally larger than that of the crystal structure (118.5 Å; Fig. 5B).

To identify the loosely folded regions in the cryo‐EM structure, the distances between the Cα atoms of each pairwise residue in the aligned structures were determined (Fig. 5C). The amino acid residues near the N‐terminal and C‐terminal ends have relatively longer Cα–Cα distances. These residues are located at the ‘domed cap’ region of the mushroom‐like structure. By contrast, the amino acid residues of the central region have relatively shorter Cα–Cα distances. They constitute the ‘stem’ region that contains extensive protomer–protomer interactions. Hence, the ‘domed cap’ region appears to be more loosely folded in the full‐length protein, as compared to the RAD52 fragment lacking the C‐terminal half.

## Discussion

In the present study, the solution structure of the human RAD52 protein was successfully determined by cryo‐EM analysis at near‐atomic resolution. The structure adopts an undecameric ring assembly. The well‐resolved part of the cryo‐EM map corresponds to the N‐terminal half of RAD52, and reveals a structure that is essentially identical to the previously reported crystal structures of the N‐terminal half. Thus, the structure of the N‐terminal half is the same, regardless of the presence or absence of the C‐terminal half. This is consistent with the previous DNA footprinting studies of ssDNA bound to the full‐length RAD52 protein or the N‐terminal fragment, which showed similar ssDNA cleavage patterns [27, 42]. The footprinting studies suggested that the structure of the ssDNA binding site is the same for the N‐terminal fragment and the full‐length protein. The present structure underscores the importance of the N‐terminal half in the oligomerisation and formation of the DNA binding site.

By contrast, the C‐terminal half of RAD52 was not visible in the cryo‐EM map, indicating that the structure is heterogeneous within the ring assembly. The C‐terminal half is more prone to proteolysis than the N‐terminal half [12], and alphafold [43] predicted that most of the C‐terminal half is intrinsically disordered. Interaction sites for RPA and RAD51, which coordinate the initial steps of homologous recombinational repair, are mapped to the C‐terminal half of RAD52. These observations suggest that the N‐terminal half plays a key structural role in oligomeric ring formation, while the C‐terminal half has a flexible structure, which may be important to enable dynamic interactions with RPA and RAD51 in DNA repair. Structural determinations of RAD52 bound to other DNA repair factors, such as RPA and RAD51, through its C‐terminal half are important to clarify the function of the C‐terminal half, and to gain a better understanding of the full‐length structure.

Previously, a heptameric ring assembly was determined by a negative staining EM analysis for the full‐length RAD52 [25]. Heptamer formation was also suggested from analytical ultracentrifugation studies [26]. Although a higher resolution structure for the heptameric assembly is required to gain detailed insight into its mechanistic role, the possibility remains that RAD52 can adopt multiple oligomerisation forms. Redβ, a functional and structural homolog of RAD52, forms multimeric rings and filaments depending on the type of DNA substrate [23]. Crystallographic studies of the N‐terminal fragment of RAD52 bound to single‐stranded DNA (ssDNA) have demonstrated that the undecameric ring form stably binds to ssDNA, and the DNA binding groove present on the surface of the ring structure appears suited for promoting the annealing reaction with the complementary DNA strand [30]. Whether the annealing reaction promoted on the surface of RAD52 involves multiple oligomerisation states awaits further structural studies.

## Conflict of interest

The authors declare no conflict of interest.

## Author contributions

WK, YT and HK designed the experiments. CK, MS and SO expressed and purified RAD52, and optimized grid preparation for cryo‐EM studies. YT collected and processed the cryo‐EM data. WK built and refined the atomic model. WK and HK guided the overall project. WK prepared the manuscript with input from all authors. All authors have given approval to the final version of the manuscript.

## Data Availability

The data that support this study are available from the corresponding authors upon request. The cryo‐EM map generated in this study has been deposited in the Electron Microscopy Data Bank (EMDB) under accession number EMD‐34430. The resulting atomic coordinates have been deposited in the Protein Data Bank (PDB) with accession number 8H1P. The atomic coordinates used in the study are available in the PDB with accession number 1KN0.
